# Bonding With Parents, Body Image, and Sociocultural Attitudes Toward Appearance as Predictors of Eating Disorders Among Young Girls

**DOI:** 10.3389/fpsyt.2021.590542

**Published:** 2021-04-13

**Authors:** Bernadetta Izydorczyk, Katarzyna Sitnik-Warchulska, Zbigniew Wajda, Sebastian Lizińczyk, Aleksandra Ściegienny

**Affiliations:** Institute of Applied Psychology, Faculty of Management and Social Communication, Jagiellonian University in Krakow, Krakow, Poland

**Keywords:** restrictive eating behavior, bulimic eating behavior, behavior toward nutrition, bonding with parents, body image, sociocultural standards attitudes toward appearance, eating disorders

## Abstract

A more holistic approach to treatment and prevention focuses on identifying the multiple risk and protective factors for eating disorders. However, there is a lack of research verifying the nature of the relationship between patterns of bonding with parents, sociocultural attitudes toward appearance, body image, and their role in developing or preventing eating disorders. The main aim of the study was to verify whether there is a specific set of risk or/vs. protective factors/measures for behaviors and dispositions related to the development of eating disorders. The study group consisted of 134 young Polish females (*M* = 14.92; SD = 1.349), with an average body mass index. The variables were measured using the Parental Bonding Instrument, the Sociocultural Attitudes Toward Appearance Questionnaire-3, The Multidimensional Body–Self Relations Questionnaire, and the Eating Disorder Inventory 3. Stepwise regression analysis was applied. Statistical analysis showed that bonding with parents (including maternal overprotection), body image (including overweight pre-occupation, fitness evaluation, health orientation, and self-classified weight), and sociocultural attitudes toward appearance (such as searching for information, pressures, and internalization) are predictors of eating disorder risks. On the other hand, maternal and paternal care (aspects of patterns of bonding with parents), positive fitness evaluation, positive appearance evaluation, and satisfaction with one's body were found to be the most significant protective factors. The results may improve prevention and intervention aimed at increasing protective factors.

## Introduction

The significant role of the Westernization phenomenon and the increased incidence of eating disorders (anorexia and bulimia) in today's adolescent population indicate the need to implement health prevention measures aimed at rapid detection of psycho-social protective factors, as well as recognition of the risk of developing eating disorders (1–3). Research done in recent years concerning problems connected with eating disorders has confirmed the significance of both gender and age as two main risk factors for developing eating disorders: such disorders are prevalent in girls and young women (4, 5). Besides gender and age, literature on the subject also lists a variety of other eating disorder risk factors: family environment, including attachment and bonding with parents; body image, including body shape dissatisfaction; low self-esteem; psychological distress; negative peer relationships; and family and media pressure (6–8).

### Family Factors Related to Eating Disorders

Family factors have been widely studied and reported in the literature in connection to the genesis, course, and treatment of eating disorders (9–11). Among the more significant aspects in that area are the relationships between children and their parents, which are most often studied on the basis of attachment theory or the parental bonding construct (12–16). Insecure attachment can be connected with a higher level of psychopathology, including eating disorders, while mediators of such a relationship very often affect dysregulation and perfectionism (14). Studies concerning the relationships between women suffering from anorexia and their parents indicate that (1) women with anorexia usually describe their parents negatively; (2) fathers are frequently perceived as inaccessible or rejecting; (3) mothers are often perceived as dominating, excessively protective (controlling), and/or perfectionists; and (4) parents are perceived as being unsupportive of their daughters' efforts to strive for independence (17). These concepts developed (18) have been used very often in studies concerning relationships between persons with eating disorders and their parents. Parental bonds that develop may be described in two dimensions, namely, care and control/overprotection; according to the authors of the abovementioned studies, the most optimal balance is a high level of care and low level of overprotection. The studies that utilized Parker, Tupling, and Brown's Parental Bonding Instrument (PBI) confirmed that model's legitimacy in reference to mental health and a wide spectrum of psychopathologies (19, 20). Additionally, the model has been applied to studies on people with eating disorders. The studies by Fichter and Quadflieg (21) were conducted on patients with anorexia and bulimia who were undergoing treatment by means of behavioral therapy (635 subjects, including 55 presented females, aged below 20); findings revealed low perceived levels of parental care and parental high levels of overprotection in subjects with bulimia, particularly in relation to fathers. The results of patients suffering from anorexia did not differ from the control group in that respect (21). Sordelli et al. (22) demonstrated differences in the way female patients with anorexia (*n* = 42) and bulimia (*n* = 26) perceived their parents. Patients with bulimia assessed their parents with high scores on both the care and protection (overprotection) scales, whereas patients with anorexia gave their parents high scores on the care scale only. The authors of those studies attributed those results as anorexic patients' tendency to idealize their parents.

Józefik et al. (23) studied bonding patterns in girls who had been diagnosed with anorexia (*n* = 40) and those diagnosed with bulimia (*n* = 32). Results revealed that parents from both groups were less emotionally involved in their relationships with their daughters than the parents from the control group (*n* = 63); at the same time, they were found to exercise more control (overprotection) over their daughters. In both study groups, as well as in the control group, maternal care was linked to higher self-esteem regarding coping in the context of social relations. In the group of girls suffering from anorexia, a lower level of paternal overprotection facilitated the girls' identification with traits that are typically considered to be feminine.

Monteleone (13) revealed a higher level of parental control (overprotection) and a lower level of parental care, as well as a higher level of childhood trauma (including maltreatment events) in the group of female patients with anorexia (*n* = 57) or bulimia (*n* = 43), in comparison with the control group (*n* = 77). Interestingly, further analyses revealed that a particularly high level of maternal control was a substantial predictor of eating disorders, but only when participants experienced lower levels of emotional abuse. Albinhac et al. (24) examined 25 young girls with anorexia, dividing them into two groups: under 14 years of age (peripubertal) and from 14 to 17 years of age (pubertal). The study also demonstrated overprotection perceived by girls from the older group. Positive correlations were noted between maternal protection and subject age when diagnosed, and between age at the time of diagnosis and paternal overprotection, as well as body mass index; a negative correlation was found between parental care and duration of illness.

Some studies have considered perceptions of parental bonding across two generations, that is, namely, the perception of bonds between girls suffering from anorexia (*n* = 43) and their parents, and the perception of bonds between those parents and their parents (the ill girls' grandparents). Anorexic patients perceived both their parents as less caring and overprotecting (controlling), more so than in the control group (*n* = 33). Maternal control and paternal care were associated with symptom severity. Maternal grandmother care was associated with eating disorder psychopathology (25).

A similar bigenerational study was conducted by Balottin et al. (12), who examined the perception of parental bonding in families with teenagers (*n* = 78) diagnosed with restricting-type anorexia, as well as the perception of bonding between those teenagers' parents and grandparents. Curiously, contrary to earlier studies, the group of girls suffering from anorexia did not differ from the control group in regard to their perception of parental bonds. However, those patients' parents, particularly their fathers, perceived their own parents as insufficiently protective, and overly controlling. As can be seen from the reports mentioned here, the information obtained varies and lacks cohesion in many aspects. Tetley's (26) systematic review of 24 studies clearly indicates that in comparison to persons without a psychiatric diagnosis, women diagnosed with eating disorders (as well as patients diagnosed with other psychiatric problems) much more frequently appraise their parental bonds as being of a low quality. The authors of that review indicate the need to conduct further studies on the relationship between perceptions of parental bonding and the development of eating disorders, as well as parents' specific behavior, which is of utmost significance in eating disorder development. The research conducted thus far shows that the insecure attachment pattern/style and abnormal bonding with parents are factors contributing to the development of eating disorders (26–29). Cortés-García et al. (5) performed a meta-analytic review of studies on how insecure attachment leads to symptoms of eating disorders, through mediation analysis. Maladaptive emotion regulation and depressive symptoms were found to be among the major pertaining factors, while body dissatisfaction, neuroticism, perfectionism, mindfulness, and social comparison were of somewhat lesser importance.

### Body Image and Sociocultural Factors Related to Eating Disorders

Sociocultural patterns and the related body image in young people are among the crucial factors in the area of eating disorders. Body image is also commonly considered to be one of important elements contributing to the development of eating disorders (30, 31). Fixed in cognitive schemas and emotions, and manifested in a person's behavior, it is defined as a psychological structure describing self-esteem and satisfaction/dissatisfaction with one's body and its parts, self-assessment of the attractiveness of one's appearance, fear of being obese (fat phobia), and/or fitness assessment (32). Body image distortions are observable in patients with eating disorders. According to the 10th revision of the International Classification of Diseases (ICD-10), anorexia nervosa can be diagnosed when an individual's body image is significantly disturbed and the disturbance leads to intrusive thoughts and anxiety about gaining weight. In the Diagnostic and Statistical Manual of Mental Disorders, Fifth Edition (DSM-5), the criterion for a disturbed body image is met when a person's self-perception of their body weight and shape is disturbed (which may include denial of a low body weight). Body image can be disturbed in various ways, such as persistent dissatisfaction with select body parts, incorrect assessment of body size or individual body parts, depersonalization of the body, body hate, and discrepancies between the ideal body image and reality. Many authors believe that internalization of a body that is too thin may be a particularly important aspect of developing eating disorders (33). Research findings have confirmed that perceived pressure to be thin and the internalization of an ideal represented by a thin person constitute a causal risk factor for body dissatisfaction, dieting, negative affect, and eating pathology (34–36). Lena et al. (37) provided some evidence that patients with eating disorders often present a distorted body image for at least 6 months before the actual diagnosis. Mountjoy et al. (38) suggested that distorted body image cognitions are the first to develop in anorexia nervosa.

Literature review pointed out the significance of the sociocultural impact of the body image standards promoted by mass media on the development of unhealthy eating behavior in different cultures. Several recent studies have confirmed that dependence. For example, Sanchez-Ruiz (39) studied the direct and indirect impact of sociocultural variables and mass media on the eating behavior of young adults (143 women and 101 men, aged 18–31 years), and the results confirmed that mass media pressure was an important predictor of this behavior. In another study (34), 514 Polish men and women were studied, and the results show that the pressure to conform to sociocultural body image and physical appearance standards had the most profound and direct influence on the development of restrictive eating behavior, appearing to negatively affect women's body image. Information searches on body image in mass media had the strongest and most direct impact on the development of bulimic eating behavior in/among women. Rodgers' (40) meta-analysis indicated significant effect sizes of exposure to pro-eating disorder websites on body image dissatisfaction (*d* = 41), negative affect, and diet. Furthermore, a systematic review by Ryding and Kuss (41) showed that appearance-related activity on social media was a stronger predictor of body image disturbances than social media use alone.

### The Current Study

Existing research on risk factors was mainly conducted on groups of persons with a psychiatric diagnosis of eating disorders in the context of examining family bonds and frequent instances of retrospective perception. Much more rarely has research been conducted on younger adolescents who are not suffering from eating disorders and are living with their parents and influenced by ongoing child–parent interactions. Studies conducted in that field have applied various and diverse measurement methods and different criteria concerning study groups, which, on the one hand, provide a comprehensive picture of the issue, but, on the other hand, render comparisons of results difficult.

Moreover, there are only incidental studies that deal with the complex measurement of psychological and sociocultural variables (potential risk factors for developing eating disorders), and these were conducted on the same population of female teenagers. The multifaceted and multi-conditional nature of eating disorders in adolescence requires that researchers focus their studies on multiple clinical and non-clinical trials to allow for superior result assessment accuracy and more apt conclusions based on the complex measurement of psychological and sociocultural variables. Due to the great importance of preventive healthcare for different determinants of eating disorders, there is a need for constant empirical research in different populations and cultures (especially undiagnosed adolescents) in order to answer questions such as whether and how individual, family, and sociocultural factors directly impact the development of unhealthy eating behavior and eating disorders.

### Research Objective, Variables, and Research Questions

Given the significance of the factors described above for the development and course of eating disorders, a study was designed in the non-clinical group with the aim of determining the power of the influence exerted by particular factors (relationship with parents, body image, and culture-related factors) upon the manifestation of behaviors connected with the risk of developing eating disorders. It is assumed that not all of them will be risk factors; on the contrary, they may also constitute protective factors/measures of eating disorders.

In the study model, the authors determined the following independent variables:

- patterns of bonding with parents—a variable that includes teenagers' subjective assessments concerning their relationships with their mothers and fathers, depicted using two dimensions: experience of care (from emotional coldness to emotional warmth) and control (from psychological autonomy to excessive parental influence and control/overprotection)
- body image
- sociocultural attitudes toward the body, including internalization (as the intensity of conscious and unconscious absorption of sociocultural body image and appearance standards promoted by mass media), pressure to conform to sociocultural body image standards (the degree of pressure a person feels and declares), and searching for information (the frequency of information searches on relevant sociocultural norms and body image and physical appearance standards promoted by the mass media).

As the dependent variable, the authors assumed a theoretical construct composed of dispositions and behavior types indicated in the literature, depicting the risk of developing eating disorders in adolescence; these were:

- nutrition-related behavior (restrictive, bulimic, perfectionist, and/or ascetic behavior)
- psychological dispositions, including self-esteem, personal alienation, interpersonal insecurity (as the level of difficulty a person has expressing personal thoughts and feelings in the presence of other people and the strength of their tendency to self-isolate), interpersonal alienation (as an indicator describing one's degree of disappointment, alienation, and lack of trust in relationships), emotional dysregulation (as an indicator of the intensity of mood instability, impulsiveness, recklessness, anger, and the self-destruction tendency), interoceptive deficits (an indicator describing one's level of confusion in terms of accurately identifying emotional states and the stimuli that come from one's body), and maturity fears (an indicator describing the degree of a person's desire to return to the safety of childhood; it is also related to the fear of psychosexual puberty).

The study model also included the body mass index (BMI) control variable (the value of this index is calculated by dividing body weight in kilograms by height in meters squared). It is assumed that optimal body weight is maintained when the BMI-value ranges from 19.5 to 24.5, with values below the mean indicating underweight or pathological weight loss, and index values above the mean range indicating overweight.

The following research questions have been formulated on the basis of the literature:

1. Can present parental bonding status in the dimensions of care and overprotection constitute a risk factor or/vs. protective factor/measure for behaviors connected with the development of eating disorders?
2. Can various aspects of body image constitute risk factors or/vs. protective factors/measures for behaviors related to the development of eating disorders?
3. Can various aspects of sociocultural attitudes toward appearance constitute risk factors or/vs. protective factors/measures for behaviors related to the development of eating disorders?

## Materials and Methods

### Participants and Procedure

The study group composed of female teenagers was formed using non-probability sampling. The following inclusion criteria were applied; they were verified *via* questionnaire items that referred directly to indices:

- age (13–19 years); Polish nationality; in primary, junior secondary, or secondary school. The age criterion was connected with the period of increased risk of anorexia and/or bulimia nervosa in adolescent girls.
- having both parents, who have been bringing up the teenager since birth and are still involved at the time of the study. Subjects from divorced and incomplete families were included, but in such cases, an inclusion criterion stipulating both the mother's and the father's participation in bringing up the subject (i.e., regular personal contact with the teenager, parents communicate regularly about the teenager's upbringing and care, etc.) was applied.
- the teenagers have never undergone pharmacological treatment and/or never participated in individual or group psychotherapy due to manifested psychic disturbances [e.g., anxiety, depression, post-traumatic stress disorder (PTSD), obsessive–compulsive issues, or others]; have never suffered from, been diagnosed with, or been treated for eating disorders (anorexia, bulimia, and compulsive overeating) at the time of joining the study; and are not visibly handicapped or physically deformed.

The study was conducted in 2018, in two primary schools, two junior secondary schools, and one secondary school in two towns located in southern Poland. Qualified researchers trained in conducting psychological research (graduate students in psychology, under supervision) conducted the research in person. This study's researchers (clinical psychologists) trained them regarding the procedures and ethics of conducting the research. Before the study, consent had been obtained from each teenager's parent/guardian as well as the teenager herself. Participants were informed that participation in the study was voluntary and anonymous. In developing the research model, the authors estimated the minimum size of the studied sample (*N* = 385). It was planned on the study design stage. However, there were restrictions imposed by the institutions where the authors were conducting the study. Due to incomplete questionnaires, some people were excluded from the research and further statistical analyses. The final study sample comprised 134 teenagers. The average age of the subjects in our study was 14.92 ± 1.34. To increase the size of the studied group, we decided to include one subject aged 20 years (a secondary school student) (Table 1).

**Table 1:** Selected characteristics of the study group (*n* = 134).

	***M***	**Me**	**Min**	**Max**	**SD**
Age	14.92	15.00	13.00	20.00	1.349
Height	164.81	164.00	151.00	178.00	5.903
Weight	54.92	53.00	38.60	103.00	10.245
BMI	20.31	19.60	15.50	46.80	4.024

*M, mean; Me, median; Min, minimum value; Max, maximum value; SD, standard deviation*.

### Compliance With Ethical Standards

Ethical approval was obtained from the relevant institutional ethical review committees, and the research was conducted in accordance with national and international regulations and guidelines. All subjects gave their informed consent before they participated in the study. The study was conducted in accordance with the Declaration of Helsinki, and the protocol was approved by the Ethics Committee of the Institute of Applied Psychology, Jagiellonian University, Krakow.

### Methods

#### The Parental Bonding Instrument PBI

Agnieszka Popiel's (42) Polish adaptation of Parker, Turbin, and Brown's (18, 20). Parental Bonding Instrument was used to measure the variable perception of bonding with parents. The instrument consists of 25 items (separate items for the mother and the father) that prompt subjects to describe their parental bonds in two dimensions: care (12 items) and control (overprotection) (13 items). According to the tool developers, high levels of care and low levels of control/protection appear to be the most optimal. The psychometric coefficient values were satisfactory in the study reported here: Cronbach's alpha coefficient: mother—care: 0.820, mother—control/protection: 0.807; father—care: 0.885, father—control/protection: 0.763.

#### The Multidimensional Body-Self Relations Questionnaire MBSRQ

For studying body image, this study utilized the MBSRQ developed by Thomas F. Cash (43, 44); it is composed of 69 items assessing subjects' emotional and cognitive self-perceived body image. The MBSRQ contains nine scales: Self-Classified Weight (SCW), Appearance Orientation (AO), Appearance Evaluation (AE), Body Areas Satisfaction Scale (BAS), Overweight Pre-occupation (OP), Fitness Orientation (FO), Fitness Evaluation (FE), Health Evaluation (HE), Health Orientation (HO), and Illness Orientation (IO). In this study, subjects responded to 69 items concerning various areas of the body; their responses were scored on a five-point Likert-type scale. In order to assess an individual's body image by means of the MBSRQ, the mean result for each scale should be appraised. The higher the result, the more satisfied the person is with their body, body parts, and bodily functions. In a Polish study (45) on a population sample of 341 women aged 18–35 years, exploratory factor analysis revealed that the Polish MBSRQ's factor structure is similar to the original version's. Its internal reliability/consistency was assessed by means of McDonald's *x* coefficient, which ranged from 0.66 to 0.91. The Polish version of the MBSRQ was translated from English to Polish by Schier using a standard forward–backward translation procedure (46).

#### The Sociocultural Attitudes Towards Appearance Questionnaire 3 SATAQ-3

We used Izydorczyk and Lizińczyk's (47) Polish adaptation of Thompson et al.'s (48) SATAQ-3. The original version consists of the following four scales: Internalization—General (a nine-item scale describing the degree of internalization of sociocultural body image standards), Internalization—Athlete (a five-item scale for measuring the degree of internalization of an athletic body shape), Pressures (a seven-item scale describing the intensity of pressure a person feels to conform to sociocultural standards), and Information (a nine-item scale that describes the frequency at which a person seeks information about body image and sociocultural standards of physical appearance). In this study, each subject completed the SATAQ-3 questionnaire by marking their answers on a five-point Likert-type scale. The Cronbach's alpha coefficients for the scales were as follows: Internalization—General = 0.93 (Polish version = 0.91), Internalization—Athlete = 0.80 (Polish version = 0.96), Pressures = 0.92 (Polish version = 0.78), and Information = 0.96 (Polish version = 0.89).

#### The Eating Disorder Inventory 3 EDI-3

We used David Garner's EDI-3 (49), which consists of 91 items organized into 12 primary scales: Drive for Thinness, Bulimia, Body Dissatisfaction, Low Self-Esteem, Personal Alienation, Interpersonal Insecurity, Interpersonal Alienation, Interoceptive Deficits, Emotional Dysregulation, Perfectionism, Asceticism, and Maturity Fears. The following three scales from the EDI-3 were used to measure the dependent variable: Body Dissatisfaction (BD), Drive for Thinness (DT), and Bulimia (B). All scales have high rates of statistical validity and reliability in Polish studies. Cronbach's alpha values for the EDI-3 scales were as follows: DS = 0.86; B = 0.81, 0.86; and BD = 0.92 (50).

#### Socio-Demographic Data

For collecting socio-demographic and autobiographical data, a questionnaire was used, with questions regarding age, nationality, place of residence, school, family of origin, diseases, disorders, disabilities, body deformations, and pharmacological and psychotherapeutic treatment received. BMI was also calculated.

### Data Analyses

Statistical analyses were performed using Statistica 13.3 and Microsoft Excel (Microsoft Office 365 ProPlus). The strength of the dependence between the dependent and independent variables was measured by means of stepwise regression analysis. The aim at this stage was to look for predictors of dependent variables in groups of Polish adolescents.

In the study reported here, it was assumed that risk factors will be those variables that turn out to be statistically significant predictors (at least *p* < 0.05) of behavior related to the development of eating disorders, for which the β factor will have a positive value. On the contrary, protective factors/measures will be those variables that turn out to be statistically significant predictors (at least *p* < 0.05) of behavior related to the development of eating disorders, for which the β factor will have a negative value.

## Results

The sample consisted of 134 adolescent girls aged 14.92 ± 1.34. The subjects' age range was similar to that of other studies [see (51, 52)]. Average BMI scores were also within the expected norms according to age. Descriptive statistics for the research variables are presented in Table 2.

**Table 2:** Selected characteristics of the study variables.

	***M***	**Me**	**Min**	**Max**	**SD**
Maternal care	27.78	29.00	10.00	38.00	6.26
Maternal overprotection	12.95	12.00	0.00	33.00	5.92
Paternal care	25.26	26.00	2.00	37.00	7.68
Paternal overprotection	10.73	10.00	0.00	28.00	5.55
Appearance orientation	44.27	45.00	23.00	60.00	7.24
Fitness evaluation	17.77	19.00	5.00	25.00	4.83
Fitness orientation	39.16	39.00	20.00	52.00	7.03
Appearance evaluation	23.57	24.00	10.00	35.00	5.72
Health evaluation	19.23	19.00	14.00	24.00	2.00
Health orientation	37.21	37.00	25.00	51.00	4.70
Overweight pre-occupation	9.99	9.00	3.00	20.00	3.79
Self-classified weight	6.01	6.00	0.00	10.00	1.67
Body areas satisfaction scale	29.32	31.00	0.00	45.00	9.17
Internalization—general	11.28	10.00	4.00	28.00	4.95
Pressures	26.05	23.00	11.00	60.00	12.00
Internalization—athlete	10.18	10.00	3.00	20.00	4.40
Information	15.93	15.50	5.00	28.00	4.88
Drive for thinness	10.99	10.00	1.00	28.00	7.21
Bulimia	3.56	3.00	0.00	19.00	3.55
Body dissatisfaction	15.97	16.00	5.00	31.00	4.28
Maturity fears	14.16	14.00	0.00	28.00	5.09
Interoceptive deficits	14.69	14.00	0.00	31.00	7.74
Low self-esteem	8.23	7.00	0.00	24.00	5.47
Personal alienation	10.66	10.00	1.00	28.00	5.68
Interpersonal insecurity	10.28	10.00	0.00	28.00	5.67
Interpersonal alienation	12.53	12.00	0.00	27.00	4.64
Emotional dysregulation	7.80	7.00	0.00	24.00	5.56
Perfectionism	7.46	7.00	0.00	18.00	4.43
Asceticism	8.45	8.00	0.00	22.00	4.74

*M, mean; Me, median; Min, minimum value; Max, maximum value; SD, standard deviation*.

Values obtained through stepwise/progressive regression analysis are presented in Table 3. The data from Table 3 demonstrate that all three independent variables, namely, bonding with parents, body image, and sociocultural attitudes toward appearance, are predictors of eating disorder risks. The most frequently encountered predictor of the dependent variable (for eight aspects of eating disorders) was bonding with parents, particularly paternal care (for five aspects of eating disorders). In 17 instances, the β factor had a negative value, allowing those aspects of the independent variables to be treated as protective measures against eating disorders.

**Table 3:** Bonding with parents (PBI), body image (MBSRQ), and sociocultural attitudes toward appearance (SATAQ-3) as predictors of protective measures and risk factors for development of eating disorders (EDI-3) (*n* = 134).

**Dependent variables (measured using EDI-3)**	**Independent variables (measured using PBI, MBSRQ, and SATAQ-3)**		**Adjusted *R^2^***
	**Standardized factors** **β**		***F* statistics**
Drive for thinness	PBI	Paternal care: *B* = −0.274^***^	*R*^2^ = 0.725; *F*_(10.107)_ = 31.48; *p* < 0.001
	MBSRQ	Body areas satisfaction scale: *B* = −0.159	
		Overweight pre-occupation: *B* = 0.519^***^	
	SATAQ-3	Pressures: *B* = 0.188^**^	
		Internalization—athlete: *B* = 0.115	
Bulimia	PBI	-	*R*^2^ = 0.200; *F*_(8.109)_ = 4.66; *p* < 0.001
	MBSRQ	Body areas satisfaction scale: *B* = −0.260^**^	
	SATAQ-3	-	
Low self-esteem	PBI	Paternal care: *B* = −0.304^***^	*R*^2^ = 0.503; *F*_(8.109)_ = 15.83; *p* < 0.001
	MBSRQ	Appearance evaluation: *B* = −0.266^**^	
		Fitness Evaluation: *B* = −0.238^**^	
	SATAQ-3	-	
Personal alienation	PBI	Paternal care: *B* = −0.238^***^	*R*^2^ = 0.503; *F*_(8.109)_ = 15.83; *p* < 0.001
		Maternal care: *B* = −0.173^*^	
		Maternal overprotection: *B* = 0.169^*^	
	MBSRQ	Overweight pre-occupation: *B* = 0.169^*^	
		Appearance evaluation: *B* = −0.199^*^	
	SATAQ-3	-	
Interpersonal insecurity	PBI	-	*R*^2^ = 0.219; *F*_(7.110)_ = 5.68; *p* < 0.001
	MBSRQ	Appearance evaluation: *B* = −0.280^***^	
	SATAQ-3	-	
Interpersonal alienation	PBI	Paternal care: *B* = −0.380^***^	*R*^2^ = 0.316; *F*_(8.109)_ = 7.76; *p* < 0.001
		Maternal care: *B* = −0.221^*^	
	MBSRQ	Fitness orientation: *B* = 0.185^*^	
	SATAQ-3	-	
Interoceptive deficits	PBI	Paternal care: *B* = −0.359^***^	*R*^2^ = 0.479; *F*_(7.110)_ = 16.40; *p* < 0.001
		Maternal overprotection: *B* = 0.246^**^	
	MBSRQ	Overweight pre-occupation: *B* = 0.297^***^	
	SATAQ-3	Information: *B* = 0.187^**^	
Emotional dysregulation	PBI	Paternal care: *B* = −0.250^**^	*R*^2^ = 0.333; *F*_(8.109)_ = 8.30; *p* < 0.001
		Maternal overprotection: *B* = 0.228^**^	
	MBSRQ	Overweight pre-occupation: *B* = 0.272^**^	
	SATAQ-3	-	
Perfectionism	PBI	Maternal overprotection: *B* = 0.342^***^	*R*^2^ = 0.376; *F*_(8.109)_ = 9.80; *p* < 0.001
	MBSRQ	Body areas satisfaction scale: *B* = −0.227^**^	
		Fitness evaluation: *B* = 0.258^**^	
		Health orientation: *B* = 0.212^**^	
	SATAQ-3	-	
Asceticism	PBI	Maternal overprotection: *B* = 0.214^**^	*R*^2^ = 0.520; *F*_(10.107)_ = 13.68; *p* < 0.001
		Paternal care: *B* = −0.193^**^	
		Maternal care: *B* = −0.179^*^	
	MBSRQ	Self-classified weight: *B* = 0.167^*^	
		Overweight pre-occupation: *B* = 0.305^***^	
		Fitness evaluation: *B* = 0.196^**^	
		Body areas satisfaction scale: *B* = −0.166^*^	
	SATAQ-3	-	
Maturity fears	PBI	-	*R*^2^ = 0.222; *F*_(8.109)_ = 5.18; *p* < 0.001
	MBSRQ	Body areas satisfaction scale: *B* = −0.267^**^	
		Appearance orientation: *B* = −0.233^**^	
		Fitness orientation: *B*= 0.236^**^	
		Health evaluation: *B* = 0.178^*^	
	SATAQ-3	-	

*MBSRQ, The Multidimensional Body–Self Relations Questionnaire; EDI-3, The Eating Disorders Inventory 3; PBI, Parental Bonding Instrument; SATAQ-3, The Sociocultural Attitudes Toward Appearance Questionnaire 3*.

* *p < 0.05*;

** *p < 0.01*;

*** *p < 0.001*.

As many as seven aspects of the independent variable influenced the Asceticism aspect of the dependent variable. For parental bonding, they were Maternal Care (*B* = −0.179), paternal care (*B* = −0.193), and Maternal Overprotection (*B* = 0.214); for body image, they were Self-Classified Weight (*B* = 0.167), Overweight Pre-occupation (*B* = 0.305), Body Areas Satisfaction scale (*B* = −0.166), and Fitness Evaluation (*B* = 0.196). The joint influence of those factors provides an explanation for about 52% of the dependent variable variance (Asceticism) (adjusted *R*^2^ = 0.520). On the other hand, there was only one case where no influence of the independent variables upon the dependent variable was noted, that is, for Body Dissatisfaction. The β factor had the highest value for prediction of Overweight Pre-occupation by Drive for Thinness (*B* = 0.519). The highest percentage of variance (72.5%) is explained by independent variables in relation to the dependent variable Drive for Thinness (adjusted *R*^2^ = 0.725).

For the sake of clarity, in Table 4, there are individual aspects that turned out to be predictive of various behaviors and bring about risks of eating disorders.

**Table 4:** The summary of protective and risk factors for the development of eating disorders.

**Independent variable predictors with a negative value**	**Dependent variable measured using eating disorders inventory 3**	**Independent variable predictors with positive a value**
**Risk factors**	**Behaviors connected to eating disorders**	**Protective factors**
Overweight pre-occupation	Drive for thinness	Body areas satisfaction scale
Pressures		Paternal care
Internalization—athlete		
-	Bulimia	Body areas satisfaction scale
-	Low self-esteem	Paternal care
		Appearance evaluation
		Fitness evaluation
Maternal overprotection	Personal alienation	Paternal care
Overweight pre-occupation		Maternal care
	Interpersonal insecurity	Appearance evaluation
Fitness orientation	Interpersonal alienation	Paternal care
		Maternal care
Maternal overprotection	Interoceptive deficits	Paternal care
Overweight pre-occupation		
Information		
Maternal overprotection	Emotional dysregulation	Paternal care
Overweight pre-occupation		
Maternal overprotection	Perfectionism	Body areas satisfaction scale
Fitness evaluation		
Health orientation		
Maternal overprotection	Asceticism	Paternal care
Self-classified weight		
Overweight pre-occupation		Maternal care
Fitness evaluation		Body areas satisfaction scale
Fitness orientation	Maturity fears	Body areas satisfaction scale
Health evaluation		Appearance orientation

## Discussion

The studied sample included adolescents who have two parents that have been bringing them up since birth and were still involved at the time of the study. There were 12 subjects from divorced or incomplete families. However, as previously mentioned, these adolescents' parents were still participating in their upbringing (i.e., regular personal contact with the teenager and regular communication between parents regarding the teenager's upbringing and care). Moreover, all these subjects were brought up from childhood to the beginning of adolescence by two cohabitating parents. From a psychological perspective, the experiences in the formative years are crucial to the development of social skills, personality, cognitive skills, thinking skills, decision making, the ability to concentrate, and behavior. According to attachment theory and the parental bonding construct, bonds with parents are formed in early childhood (mainly during parent–infant interaction) [see (12, 53)]. Therefore, all subjects grew up in similar households during childhood.

The study revealed several predictors of behavior related to eating disorders. In 20 cases, those predictors had a negative value, which means that they can be treated as protective factors/measures, whereas in 19 cases, the β factor had a positive value, thus constituting risk factors for eating disorders. Maternal and paternal care, appearance evaluation, and body areas satisfaction, may be considered protective measures/factors. The following turned out to be risk factors: maternal overprotection, self-classified weight (SCW), overweight pre-occupation (OP), fitness orientation (FO), health evaluation (HE), health orientation (HO), internalization of an athletic body type, pressure to conform to body image standards, and information searches on body image in mass media.

### Family Predictors of Eating Disorders

Maternal and paternal care turned out to be protective factors, whereas maternal overprotection was found to be a risk factor. Paternal overprotection was a neutral predictor for development of eating disorders, with neither a plus nor a minus sign. Curiously, a high level of paternal care appeared to be associated with a reduced eating disorder risk in as many as seven areas, namely, Drive for Thinness, Low Self-Esteem, Personal Alienation, Interpersonal Alienation, Interoceptive Deficits, Emotional Dysregulation, and Asceticism. That finding is interesting because, first of all, paternal care influences more risk factors related to eating disorders than maternal care, which only influences three areas, and secondly, many studies using PBI that have been conducted so far have stressed a low level of maternal or paternal care as a coexisting or risk factor of eating disorders rather than as a protective factor (26). In recent studies (13) in which similar tools were applied (the PBI and EDI-3), parental care showed no significant correlation with the areas investigated by means of the EDI-3 in the group of persons suffering from eating disorders. However, in the control group of healthy women, it correlated inversely with two aspects of the risk of developing eating disorders: perfectionism and interpersonal distrust. The substantial differences between the study reported here and other studies that have been conducted so far concern the non-clinical study group as well as the subjects' young age (mean age: 15 years). Perhaps at a younger age, such aspects as self-esteem, drive for thinness, or others constituting risk factors for developing eating disorders matter more in healthy/non-suffering girls. In that case, subjects' age also constitutes a methodological aspect: the subjects assessed their parental bonding in their present lives and not in the past, as the participants were girls who were still living with their parents and being brought up by them. The instructions for using the PBI recommend assessing parents' behavior for subjects under 16 years of age, whereas in many existing studies, the subjects were over 18 years of age. Another aspect may be that of the changing (increasing in prominence) role of the father in children's upbringing compared with previous years. It is now known that the father's role is of the utmost importance in building teenagers' identity and sense of value (54, 55). In but a few aspects, parental overprotection was a predictor of eating disorder risks, while maternal overprotection appeared to influence such aspects as Personal Alienation, Interoceptive Deficits, Emotional Dysregulation, Perfectionism, and Asceticism. Paternal overprotection was not a predictor for aspects measured by the EDI-3. In a majority of studies that have use the PBI, maternal and paternal overprotection were either a coexisting factor or a risk factor for developing eating disorders, especially in studies on female patients with binge-eating episodes and generalized nutrition problems (26). It is interesting that parental bonding, in both the care and overprotection dimensions, was not found to have a significantly strong relationship with bulimic behavior. The result appears intriguing, as in numerous other studies, the relationships between those dimensions and bulimia and impulsiveness were indicated (56–58). One of the explanations of that phenomenon may be the use of the EDI-3 questionnaire for measuring bulimic behavior, in that the questionnaire differentiates between bulimic disposition and behavior, and emotional dysregulation, interoceptive deficits, and generally understood impulsive behavior. It is worthwhile to refer again to Fassino (56), who has been demonstrated that the paternal care pattern correlates with interoceptive deficits and impulsiveness.

### Body Image and Eating Disorders

The literature indicates that body image is grossly distorted in eating disorders (30–32). Some studies indicate the role of sexual orientation in the relationship between body perception and eating psychopathology (59). However, such studies focused on adults who have usually identified a pattern of sexual orientation. The average age of the subjects in our study was 14.92 ± 1.34. Adolescents are dynamic and heterogeneous in how they define and experience their sexual orientation (60). Moreover, distorted body image in people with diagnosed mental disorders does not automatically imply the same status before the disorders emerged; much less attention is paid to this in the literature. However, we assumed that, in our study, various aspects of body image will be risk factors for developing eating disorders in young girls that had not been diagnosed with that disorder. Those expectations were confirmed, with Overweight Pre-occupation scale having the highest prediction coefficient, in comparison with Drive for Thinness and Asceticism. The Overweight Pre-occupation scale shows the degree of fear of obesity, frequency of body mass monitoring, and the tendency to follow various diets (61); as our study indicates, this may be a high risk factor for involvement in behavior characteristic of anorexia. Overweight Pre-occupation was also a predictor of Personal Alienation, Interoceptive Deficits, and Emotional Dysregulation. Risk factors in the body image area for various aspects of eating disorders also included Appearance Orientation, Fitness Orientation, Health Evaluation, and Health Orientation. Interestingly, the last three scales listed above are health-promoting ones: those who score high on those scales care about their physical fitness, integrate physical activities into their lifestyle, and are highly conscious of health-related factors. This indicated the need to carry out further more detailed studies in that respect and use intuition when organizing health-related prevention campaigns to achieve an optimal balance, as narrowly oriented promotion of dieting and physical exercise could increase the risk of behaviors connected with eating disorders.

Among the body image aspects that the results suggest could be protective factors, the following three demonstrated predictive value: the Body Areas Satisfaction scale (the higher one's satisfaction with one's body, the lower the score on the EDI-3 scales measuring Drive for Thinness, Bulimia, Perfectionism, Asceticism, and Maturity Fears), Appearance Evaluation (the higher one's satisfaction with one's appearance, the higher one's self-esteem and the lower the Personal Alienation and Interpersonal Insecurity), and Appearance Orientation, which was found to influence Maturity Fears. Thus, it is important to work on those aspects, particularly in the context of universal prevention. The results for Fitness Evaluation are interesting but ambiguous, given the negative β factor for Low Self-Esteem and the positive one for Perfectionism and Asceticism. It is likely that the variable may be a protective factor only for a selected group of people who have peculiar characteristics, such as low self-esteem without the inclination toward perfectionism or ascetic behavior; however, such a hypothesis would require further research. Treating body image as a protective factor may be particularly significant for universal education and prevention among children and early adolescent youths. The well-known prevention approach aimed at reducing risk factors, particularly in late adolescence, and at dealing with adults and groups who have particular risks, may not be as effective for younger people who have not yet manifested any disturbance in that sphere (62). Working on body image may be particularly important when moving from childhood to adolescence; during this transition, physical bodily changes are extremely conspicuous, and young people have to “reorganize” their attitude to their body (63).

### Sociocultural Predictors of Eating Disorders

The third independent variable, sociocultural attitudes toward appearance, had the lowest predictive value. In this case, risk factors include internalization of an athletic body type (Internalization Athlete) and pressures (Pressures), which are predictors of more intense Drive for Thinness, as well as frequent information searches on body image in mass media (Information), which turned out to be a predictor of interoceptive deficits (Interoceptive Deficits scale). Although the influence of sociocultural factors on eating disorders has been thoroughly described and documented (31, 35, 40), it did not manifest in our study to the expected degree. Our study revealed no significant score on the Internalization—General scale that would have been evidence of general internalization of sociocultural norms concerning appearance. In previous studies conducted in Poland, general internalization of sociocultural norms concerning appearance and information searches on body image in mass media directly influenced bulimic behavior, but only in men, whereas pressures influenced restrictive behavior (34). In that scope, the influence of sociocultural pressure on behaviors aimed at body thinning was confirmed (64, 65). The partial discrepancies concerning the results could be explained by the study sample characteristics; as in the studies of Izydorczyk et al. (34), the majority of participants were women between 19 and 35 years of age, and young men between 16 and 18 years. On the other hand, a study conducted on a similar group of subjects (teenage girls and boys between 14 and 19 years of age) indicated that mass media consumption, especially watching television shows, correlated with dysfunctional eating behavior among females. Thus, the results we obtained are not univocal and difficult to compare with previous studies. The differences may be due to study group characteristics and cultural factors. At the same time, studies' distinct aims should be indicated: in our study, we explored the direct influence of specific factors on risky behaviors, whereas in many other studies, the aim was to determine the mediating or moderating roles of various factors, including body image, on eating disorders (64, 66, 67). Mölber et al. (68) indicated that body image disturbance in eating disorders is characterized by an idealization of being underweight in conjunction with a high degree of body dissatisfaction rather than a visual perceptual disturbance. The effect of sociocultural influence may be indirect. Standards that have already been internalized have the same status as personal belief, thus determining attitudes toward the body and body image [see (34)].

In contemporary prevention, besides risk factors, preventive factors are substantial and need to be taken into account. In the context of eating disorders and body image, Thomas Cash et al. laid the foundation for a positive attitude to prevention by stressing the importance of various preventive measures in reference to body image (43, 69). Subsequent studies have confirmed the protective role of body image and related constructs such as self-esteem and appreciation of one's own body, self-care in response to internal signals concerning needs (e.g., hunger), and positive self-perception (70). The present study's results correspond to this need.

### Limitations and Future Directions

This study had its limitations. First of all, the sample size was relatively small, and all the subjects belonged to one gender (female). However, it must be stated that there are differences between women and men in terms of body image, and the sample size was adequate for a stepwise regression model. Choosing only girls allowed the authors to perform sufficient statistical analysis. No distinctions were made within the study group between early and late adolescence, which could demonstrate a detailed specificity of individual predictors depending on age. Secondly, the study is partly of a replicate nature in that it has not explored more complex interdependence between variables, taking into account their mediating or moderating roles. The authors focused on specific family factors (relationships with parents), personality factors (body image), and cultural factors, their interactions, and predictive roles for eating disorders. There is a lack of these kinds of studies, especially in the Polish population. In future research, it would be valuable to consider other variables, including parents' psychopathology. Longitudinal studies in larger population or groups with an eating disorder diagnosis would also be interesting for further research.

## Conclusions

The study revealed several protective and risk factors for eating disorders. The factors taken into account are often referred to in the literature as correlating with eating disorders, namely, bonding with parents, body image, and sociocultural factors; each is treated in a multifaceted manner in this study. The results of the study confirm that the set of intercorrelated factors may increase the risk of psychopathology, but some moderator variables may also change the directions of the relationships within this set of factors. The findings of the present study may contribute to developing preventive programs in the future.

## Data Availability Statement

The datasets generated for this study are available on request to the corresponding authors.

## Ethics Statement

The studies involving human participants were reviewed and approved by Ethics Committee of Institute of Applied Psychology, Jagiellonian University in Krakow. The patients/participants provided their written informed consent to participate in this study.

## Author Contributions

BI: conceptualization, project administration, supervision, and funding acquisition. BI, KS-W, and SL: data curation. BI, KS-W, ZW, and SL: formal analysis and methodology. BI, KS-W, ZW, and AŚ: resources. SL: software. AŚ: investigation. BI, KS-W, and ZW: validation, writing—original draft, and writing—review and editing. KS-W and ZW: visualization. All authors: have read and agreed to the published version of the manuscript.

## Conflict of Interest

The authors declare that the research was conducted in the absence of any commercial or financial relationships that could be construed as a potential conflict of interest.
